# Supplementary material for: Reconfigurable shape-morphing dielectric elastomers using spatially varying electric fields
Source: Nat Commun. 2019 Jan 14;10:183. doi: 10.1038/s41467-018-08094-w (PMC6331644; doi:10.1038/s41467-018-08094-w)
Supplement: Supplementary file 1 — Supplementary Information [file 41467_2018_8094_MOESM1_ESM.pdf]

Supplementary Information

Reconfigurable Shape-Morphing Dielectric Elastomers Using Spatially Varying Electric Fields

Ehsan Hajiesmaili[1] and David R. Clarke[1*]

[1]John A. Paulson School of Engineering and Applied Sciences, Harvard University, 29 Oxford Street, Cambridge, Massachusetts 02138, USA

[*]Correspondence to: clarke@seas.harvard.edu

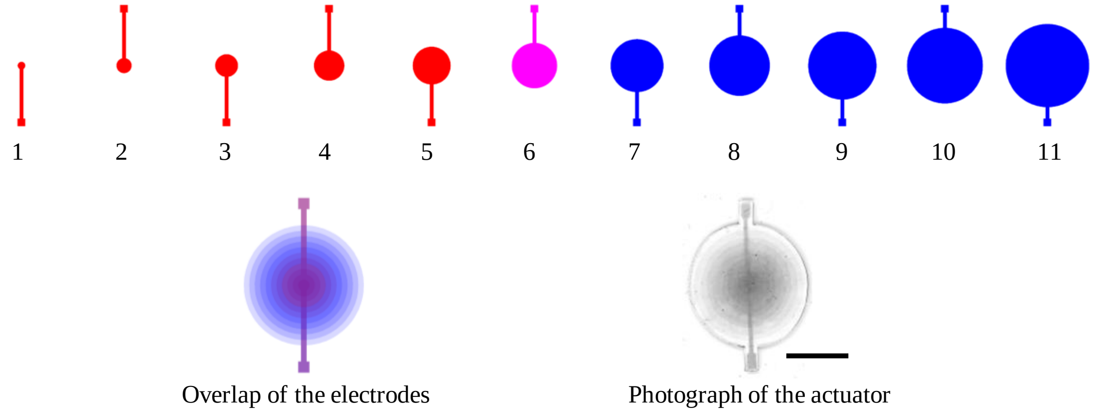

Supplementary Figure 1. To morph a flat sheet of dielectric elastomer to a positive Gaussian curvature, the meso-architecture of the electrodes consists of 11 concentric, inter-digitated electrodes of decreasing radii with height. As a result, the overlap of the electrodes increases linearly with radius. The electrodes below the mid-surface (electrodes 1-5) and above the mid-surface (electrodes 7-11) are represented by red and blue, respectively. Scale bar: 10 mm.

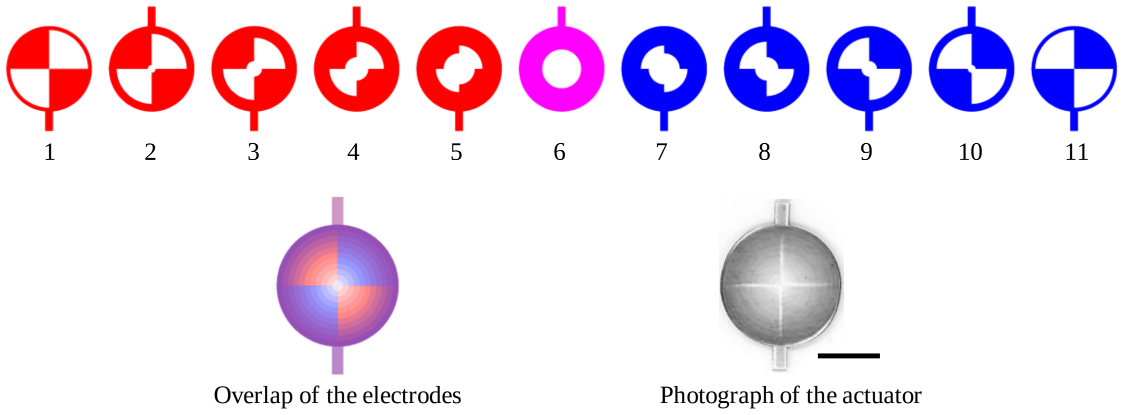

Supplementary Figure 2. To morph a flat sheet of dielectric elastomer to a negative Gaussian curvature, the meso-architecture of the electrodes consists of 11 inter-digited electrodes whose overlap increases linearly with radius, with the additional feature that on two opposite quadrants of the disk it increases with height while on the other two quadrants it decreases. The electrodes below the mid-surface (electrodes 1-5) and above the mid-surface (electrodes 7-11) are represented by red and blue, respectively. Scale bar: 10 mm.

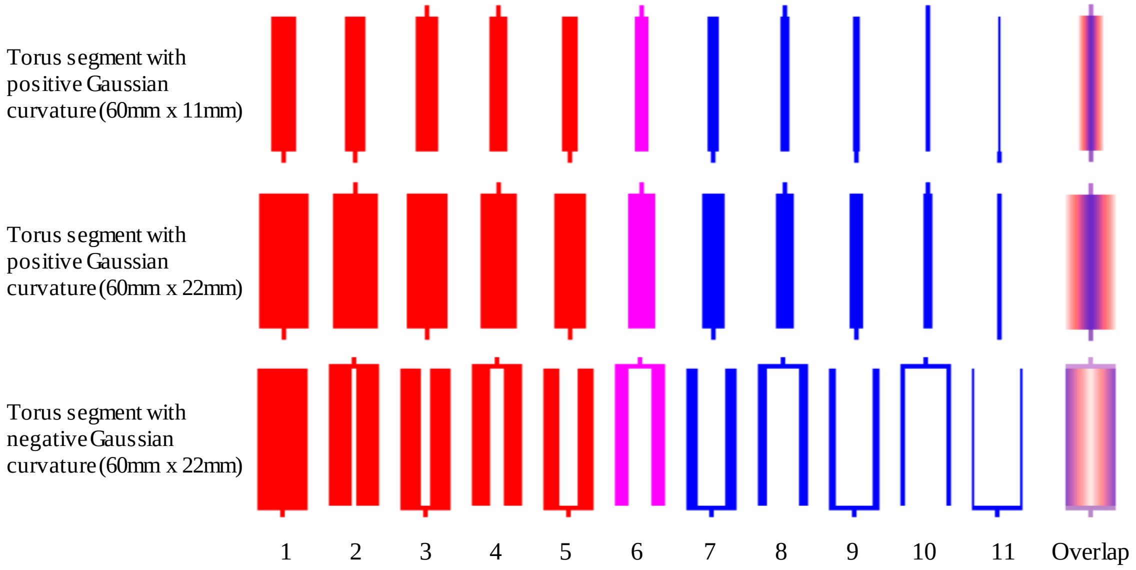

Supplementary Figure 3. Meso-architecture of the electrodes for generating torus segments with positive and negative curvatures. To morph a flat strip of dielectric elastomer into a torus segment with positive Gaussian curvatures, in the first row, the electrodes are a set of strips that are 60mm long and whose width decreases from 11mm on the bottom layer to 1mm on the top layer. The second row shows the design of the electrodes for another torus segment with positive curvature, where the electrodes are a set of strips that are 60mm long and whose width changes from 22mm on the bottom layer to 2mm on the top layer. The third row shows the electrode design for a torus segment with negative curvature. The electrodes are designed such that all the adjacent electrodes overlap at the two edges of the strip and the overlap decreases linearly to zero at the center of the strip. The electrodes below the mid-surface (electrodes 1-5) and above the mid-surface (electrodes 7-11) are represented by red and blue, respectively.

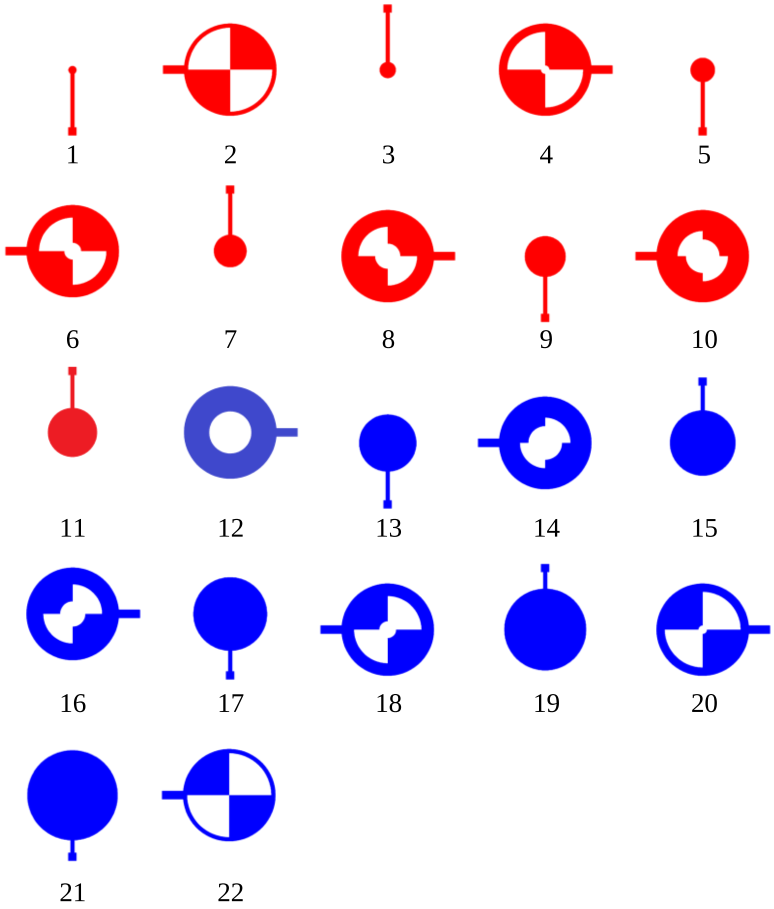

Supplementary Figure 4. The meso-architecture of the electrodes for a reconfigurable shape-morphing dielectric elastomer that morphs from a flat sheet into a dome-like shape with positive Gaussian curvature or a saddle-like shape with negative Gaussian curvature, based on which set of electrodes are addressed, The meso-architecture of the electrodes consists of alternating between the 11 electrodes of the dome-like shape and the 11-electodes of the saddle-like shape. The electrodes below the mid-surface (electrodes 1-11) and above the mid-surface (electrodes 12-22) are represented by red and blue, respectively.

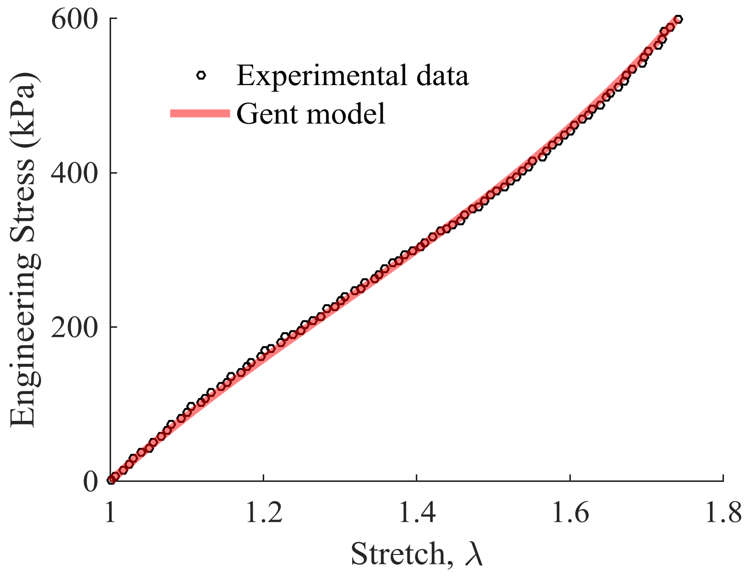

Supplementary Figure 5. Uniaxial stress-stretch curve of the elastomer (black circles) and the fitted Gent model (red line).
