# Supplementary material for: Reconfigurable shape-morphing dielectric elastomers using spatially varying electric fields
Source: Nat Commun. 2019 Jan 14;10:183. doi: 10.1038/s41467-018-08094-w (PMC6331644; doi:10.1038/s41467-018-08094-w)
Supplement: Supplementary file 3 — Description of Additional Supplementary Files [file 41467_2018_8094_MOESM3_ESM.pdf]

Description of Additional Supplementary Files

File Name: Supplementary Movie 1

Description: Real-time video recording of a thin sheet of dielectric elastomer morphing into a dome shape with positive Gaussian curvature under a sinusoidal applied voltage with peak-to-peak amplitude of 3.5 kV and period of 2 seconds. The elastomers are transparent and so a fiducial grid of black lines was marked on their surface for visualization

File Name: Supplementary Movie 2

Description: Real-time video recording of a thin sheet of dielectric elastomer morphing into a saddle shape with negative Gaussian curvature under a sinusoidal applied voltage with peak-to-peak amplitude of 3.5 kV and period of 2 seconds. The elastomers are transparent and so a fiducial grid of black lines was marked on their surface for visualization.

File Name: Supplementary Movie 3

Description: Real-time video recording of a reconfigurable thin sheet of dielectric elastomer first morphing into a dome shape with positive Gaussian curvature when the voltage is applied to a first set of electrodes and second into a saddle shape with negative Gaussian curvature as the voltage is applied to the second set of electrodes. The applied voltage was sinusoidal with peak-to-peak amplitude of 2.5 kV and period of 1 second. A fiducial grid of black lines was marked on the surface of the elastomer for visualization.
